# Secondary Haemophagocytic Lymphohistiocytosis Triggered by Adult-Onset Still's Disease in Mid-trimester Pregnancy Initially Presenting as an Upper Respiratory Tract Infection: A Case Report

**DOI:** 10.7759/cureus.104774

**Published:** 2026-03-06

**Authors:** Mst Hiramoti, Nahin Fardin Ruthi

**Affiliations:** 1 General Internal Medicine, Luton and Dunstable Hospital, Bedfordshire Hospitals NHS Foundation Trust, Dunstable, GBR

**Keywords:** adult-onset still’s disease, hyperferritinaemia, interleukin-1 inhibitor, macrophage activation syndrome, multidisciplinary management, myopericarditis, pericardial effusion, pregnancy, secondary hemophagocytic lymphohistiocytosis

## Abstract

Adult-onset Still’s disease (AOSD) is a rare systemic inflammatory disorder, characterised by fever, arthritis, and an evanescent rash. A severe complication is macrophage activation syndrome (MAS), also known as secondary haemophagocytic lymphohistiocytosis (HLH), a life-threatening state of uncontrolled immune activation that may lead to multiorgan failure. Diagnosis during pregnancy is particularly challenging because of overlapping clinical and biochemical features.

We report a 32-year-old African female primigravida at 16 weeks’ gestation, presenting with sore throat, dysphagia, chest pain, fever, rash, and progressive polyarthralgia for three weeks. The atypical presentation delayed diagnosis. Further evaluation confirmed AOSD, complicated by secondary HLH, with marked hyperferritinaemia, hypertriglyceridaemia, and persistent high inflammatory markers. Her clinical course was further complicated by pericardial effusion and suspected myopericarditis, requiring tertiary multidisciplinary care. Treatment with high-dose corticosteroids and an interleukin-1 (IL-1) antagonist resulted in clinical improvement, although she was later readmitted with an HLH flare, requiring escalation of immunosuppressive therapy.

This case highlights the diagnostic difficulty of AOSD-associated HLH in pregnancy and emphasises the importance of early recognition and timely immunosuppressive treatment to improve maternal outcomes.

## Introduction

Haemophagocytic lymphohistiocytosis (HLH) is a severe hyperinflammatory syndrome, characterised by uncontrolled immune activation, cytokine storm, and multiorgan dysfunction. HLH may be primary (genetic) or secondary to infection, autoimmune disease, malignancy, or pregnancy-associated immune dysregulation. When secondary HLH is precipitated by an underlying rheumatologic disorder, it is termed macrophage activation syndrome (MAS) [1-4].

However, adult-onset Still’s disease (AOSD) is a systemic autoinflammatory disorder, characterised by fever, rash, arthritis, hyperferritinaemia, and elevated inflammatory markers. AOSD is an uncommon condition and is generally associated with a favourable prognosis; however, severe complications, such as MAS or secondary HLH, may occur and can be life-threatening. AOSD is a recognised precipitant of secondary HLH and may present with life-threatening systemic inflammation [2,4,5]. Therefore, prompt recognition and early therapeutic intervention are essential when HLH is suspected in patients with AOSD, to reduce the risk of morbidity and mortality [6,7].

Clinical manifestations of HLH are often nonspecific, including fever, cytopenias, liver dysfunction, hypertriglyceridaemia, and hyperferritinaemia, frequently leading to delayed diagnosis [3,4]. Diagnosis of HLH during pregnancy is challenging because its clinical and laboratory features overlap with several pregnancy-related conditions, and physiological changes in pregnancy may alter laboratory parameters and mask typical findings. AOSD complicated by HLH in pregnancy is exceptionally rare, and this rarity further increases the risk of delayed recognition, as symptoms can easily be mistaken for infection or other pregnancy-associated conditions. Pregnancy further complicates recognition and treatment because of concerns regarding maternal-foetal safety of immunosuppressive therapy [8,9].

We describe a case initially presented with upper respiratory tract infection (URTI) and subsequently complicated with AOSD and secondary HLH in mid-trimester pregnancy. Later developed pericardial effusion and suspected myopericarditis, requiring tertiary multidisciplinary care.

## Case presentation

A 32-year-old African female, previously fit and well, primigravida at 16 weeks’ gestation, presented with a three-week history of severe sore throat, lethargy, odynophagia, pleuritic chest pain, fever, and progressive polyarthralgia with joint swelling, accompanied by an erythematous rash on the face and trunk, which was mildly itchy. In the community, she was treated by a GP with oral amoxicillin during the first week of her initial presentation, but her symptoms worsened, and she was unable to eat or drink in the last couple of days. Observations showed tachycardia with a heart rate (HR) of 113, blood pressure (BP) 110/78 mmHg, temperature 37.8°C, and a pain score of 10/10. On physical examination, the back of the throat showed signs of follicular pharyngitis. Musculoskeletal examination confirmed bilateral (B\L) wrist, all hand, ankle, and knee joint tenderness and swelling, with joint movements severely restricted by pain. The abdomen was mildly tender to touch. Initial laboratory evaluation demonstrated elevated inflammatory markers and mild liver enzyme derangement (Table 1). Preliminary evaluation suggested an upper respiratory tract infection (URTI), suspected epiglottitis, and reactive arthritis, followed by a recent URTI.

**Table 1:** Blood test result on admission ALP, Alkaline Phosphatase; ALT, Alanine Aminotransferase; APTT, Activated Partial Thromboplastin Time; ANA, Antinuclear Antibody; CRP, C-Reactive Protein; RBC, Red Blood Cell; WBC, White Blood Cell; MCV, Mean Corpuscular Volume; MCH, Mean Corpuscular Haemoglobin; MCHC, Mean Corpuscular Haemoglobin Concentration; RDW, Red Cell Distribution Width; tTG IgA, Tissue Transglutaminase Immunoglobulin A

Analyte	Value	Normal Range	
Sodium	133 mmol/L	133-146 mmol/L	
Potassium	3.4 mmol/L	3.5-5.3 mmol/L	
Urea	2.2 mmol/L	2.5-7.8 mmol/L	
Creatinine	53 µmol/L	44-80 µmol/L	
Total Protein	71 g/L	60-80 g/L	
Globulin	45 g/L	-	
Total Bilirubin	23 µmol/L	0-20 µmol/L	
ALP	145 U/L	30-130 U/L	
ALT	51 U/L	0-32 U/L	
Prothrombin Time	15.2 sec	9-14 sec	
INR	1.3	0.8-1.2	
APTT	27.8 sec	26-38 sec	
Calcium	2.33 mmol/L	2.2-2.6 mmol/L	
Albumin	26 g/L	35-50 g/L	
Adjusted Calcium	2.61 mmol/L	2.2-2.6 mmol/L	
CRP	181 mg/L	0-4.9 mg/L	
WBC	11.5 × 10⁹/L	4-11 × 10⁹/L	
Haemoglobin	103 g/L	120-160 g/L	
Platelets	322 × 10⁹/L	150-450 × 10⁹/L	
RBC	3.5 × 10¹²/L	4-5.2 × 10¹²/L	
Haematocrit	0.30 L/L	0.36-0.46 L/L	
MCV	85 fL	80-100 fL	
MCH	29.1 pg	27-32 pg	
MCHC	341 g/L	280-355 g/L	
RDW	14.20%	11.8-14.8%	
Neutrophils	9.44 × 10⁹/L	2-7 × 10⁹/L	
Lymphocytes	0.93 × 10⁹/L	1-3 × 10⁹/L	
Monocytes	1.13 × 10⁹/L	0.2-1 × 10⁹/L	
Eosinophils	0 × 10⁹/L	0-0.4 × 10⁹/L	
Basophils	0 × 10⁹/L	0.02-0.1 × 10⁹/L	
Rheumatoid Factor	14.6 IU/mL	0-13.9 IU/mL	
Vitamin B12	691 pg/mL	191-663 pg/mL	
Folate	14.1 µg/L	4.5-37.3 µg/L	
ANA	Negative	Negative	
Bile Acids	4 µmol/L	0-19 µmol/L	
tTG IgA	0.3 U/mL	0-3.9 U/mL	

ENT specialist assessment, including flexible naso-endoscopy, showed no evidence of epiglottitis or deep neck space infection. Extensive viral and bacterial investigations, including blood and urine cultures, were negative. Rheumatological screening, including antinuclear antibody (ANA) and rheumatoid factor (RF), was also unremarkable (Table 1).

**Figure 1:**
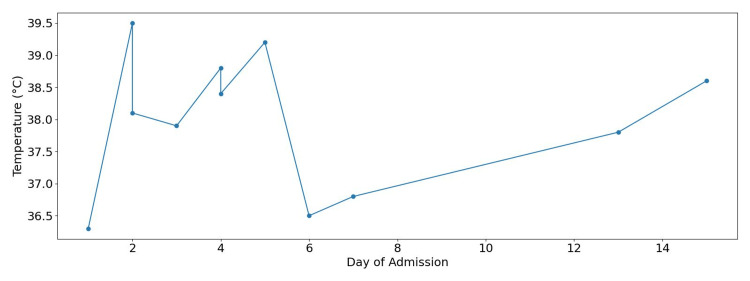
Temperature trend during admission

**Figure 2:**
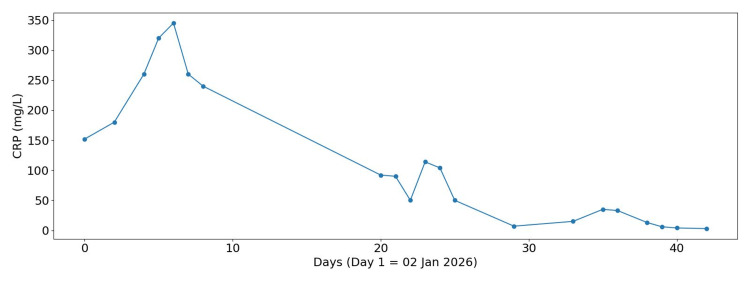
CRP trend during admission CRP, C-Reactive Protein

**Figure 3:**
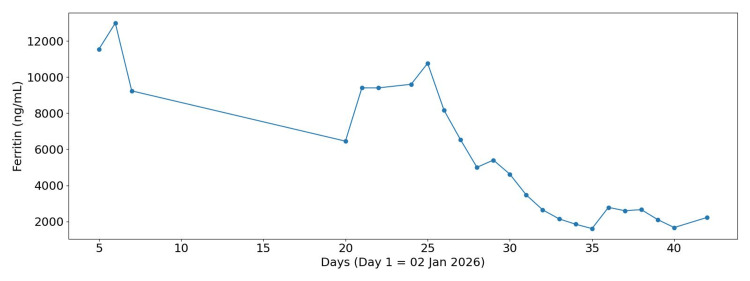
Ferritin trend during admission

Subsequent evaluation noted that, despite the patient being on broad-spectrum antibiotics (intravenous meropenem), she had persistent high temperature (Figure 1), synovitis, sore throat, rash, raised inflammatory markers (high C-reactive protein (CRP), Figure 2), and hyperferritinaemia (Figure 3), raising suspicion for AOSD, while the degree of inflammation prompted evaluation for secondary HLH, as suggested by our local rheumatology team on Day 4 of admission.

**Figure 4:**
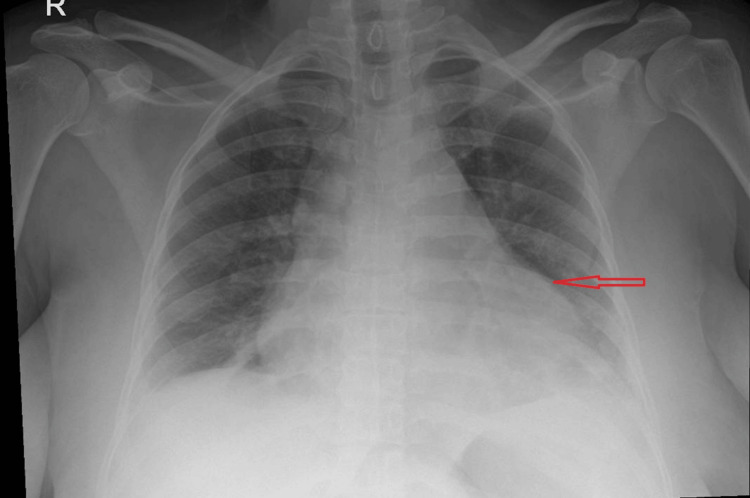
Chest X-ray (AP view) demonstrating cardiomegaly (red arrow)

**Figure 5:**
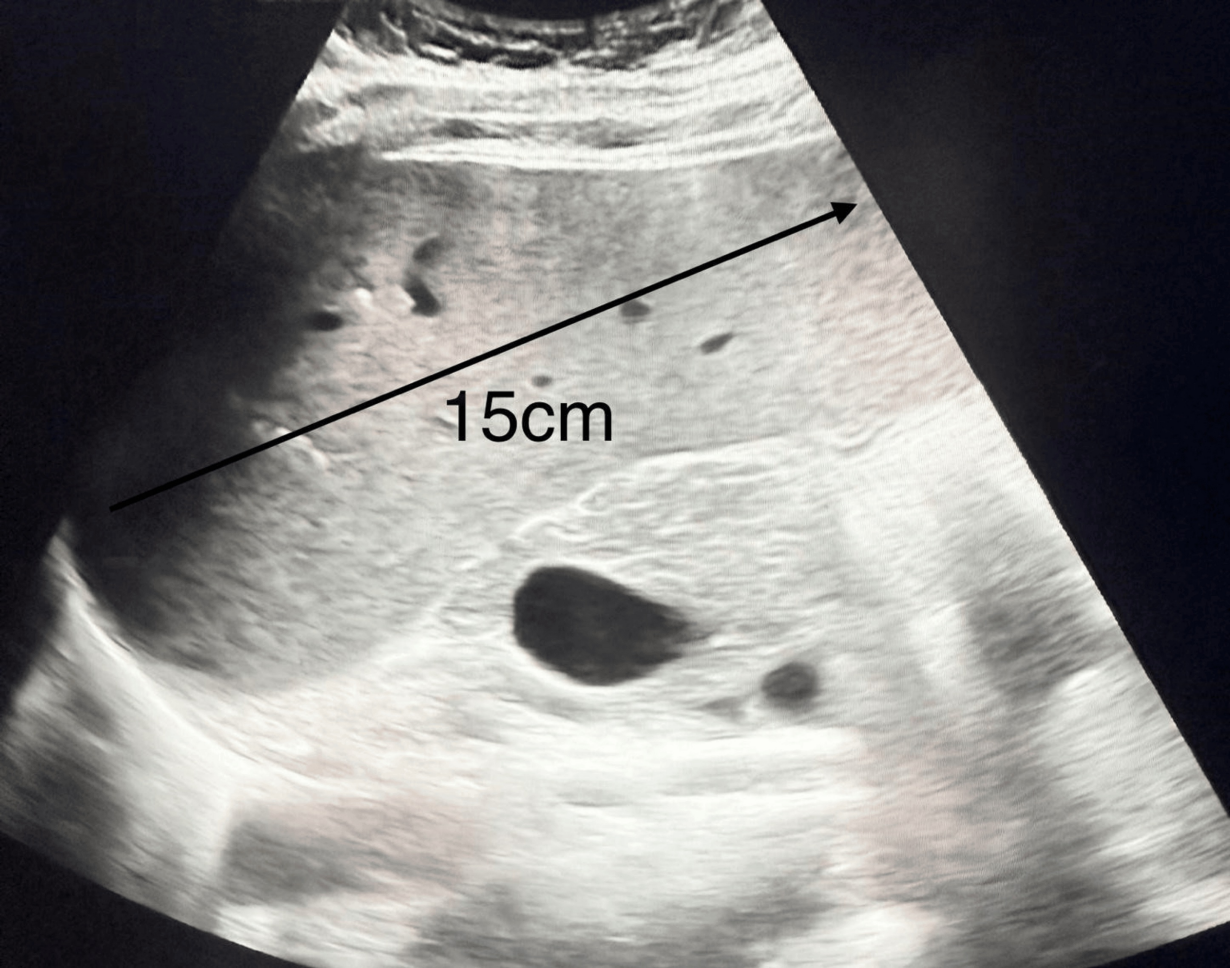
Abdominal ultrasound demonstrating moderate splenomegaly The spleen is moderately enlarged measuring 15 cm in oblique length (arrow).

Further investigations revealed marked hyperferritinaemia, elevated triglycerides, and fibrinogen (Table 2). Although fibrinogen levels are typically low in HLH due to consumption coagulopathy, they may be normal or even elevated in the early inflammatory phase because fibrinogen is an acute-phase reactant. In our case, the initially elevated fibrinogen likely reflected early-stage HLH with severe systemic inflammation [1]. In addition, persistently raised CRP (Figure 2), cardiomegaly on chest X-ray (CXR) (Figure 4), and no identifiable infectious source on Day 5 of admission were noted. Abdominal ultrasound confirmed moderate splenomegaly (Figure 5). At this stage, the differential diagnoses included AOSD and AOSD complicated by secondary HLH/MAS. Several red-flag features raised concern for evolving HLH, including persistent fever, markedly elevated ferritin levels (peak approximately 13,000 ng/mL; reference range 15-150 ng/mL), hypertriglyceridaemia, splenomegaly, and rapidly worsening systemic inflammation. 

**Table 2:** Special blood test relevant to haemophagocytic lymphohistiocytosis on Day 5

Analyte	Day 5 Value	Normal Range	
Ferritin	12,991 ng/mL	15-200 ng/mL	
Triglyceride	4.4 mmol/L	≤1.7 mmol/L	
Fibrinogen	8.4 g/L	1.5-4.0 g/L	

On the other hand, underlying infection and occult malignancy were not evident on laboratory or imaging studies. Establishing a definitive diagnosis was crucial to enable the timely initiation of treatment, as the patient’s clinical condition continued to deteriorate. The patient fulfilled the Yamaguchi diagnostic criteria for AOSD, supporting the diagnosis of AOSD. A diagnosis of AOSD requires at least five criteria in total, including a minimum of two major criteria. The major criteria consist of a fever higher than 39 °C lasting at least one week, arthralgia or arthritis persisting for two weeks or more, the presence of a typical rash, and leukocytosis with high polymorphonuclear cells. The minor criteria include a sore throat, newly developed significant lymphadenopathy, hepatomegaly or splenomegaly, abnormal liver function tests, and negative ANA (immunofluorescence assay (IFA)) and RF (immunoglobulin M (IgM)) results. Prior to applying these criteria, infections, malignancies - particularly lymphoma - and other rheumatic diseases, such as systemic vasculitis, must be excluded [9].

Concurrently, the patient also demonstrated features suggestive of MAS according to the 2004 MAS criteria [10] and the 2016 MAS classification criteria [11], further raising concern for AOSD-associated MAS. A diagnosis of HLH can be considered when at least five of the eight HLH‑2004 criteria are fulfilled, or when a molecular abnormality consistent with HLH is identified. These criteria include persistent fever, splenomegaly, and cytopenias affecting at least two blood cell lines - specifically, haemoglobin below 9 g/dL (reference range: 12-16 g/dL), platelets under 100,000/mm³ (reference range: 150,000-450,000/mm³), or neutrophils below 1,000/mm³ (reference range: 2,000-7,000/mm³). Additional criteria involve hypertriglyceridaemia, with fasting triglycerides of 265 mg/dL or higher, and/or hypofibrinogenaemia, with fibrinogen levels of 150 mg/dL or less, as well as evidence of haemophagocytosis in the bone marrow, spleen, lymph nodes, or liver without an underlying malignancy to explain it. Low or absent NK‑cell activity, ferritin levels of at least 500 ng/mL, and elevated soluble IL‑2 receptor (sCD25) concentrations of 2,400 U/mL or higher also contribute to meeting the diagnostic threshold [10,11].

Due to strong suspicion for secondary HLH and treatment with systemic corticosteroids, intravenous methylprednisolone, followed by anakinra, was initiated on Day 6 of admission, after the multidisciplinary team discussion involving obstetrics and rheumatology teams from both the local and tertiary centres.

**Figure 6:**
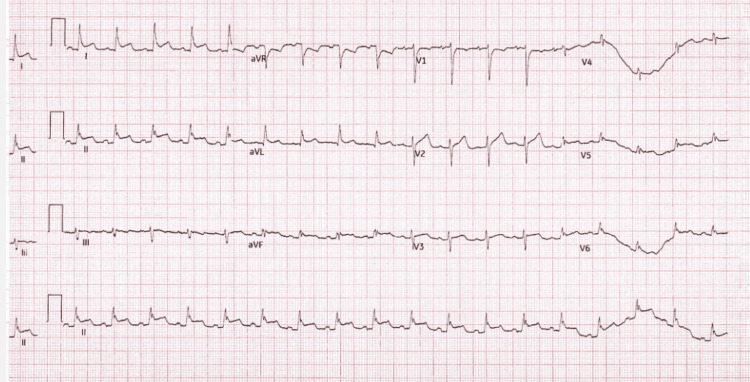
ECG demonstrating mild widespread ST elevation ECG, Electrocardiographic

**Figure 7:**
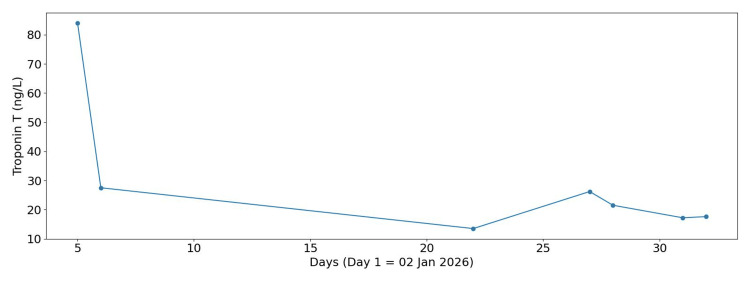
Troponin trend during admission

**Figure 8:**
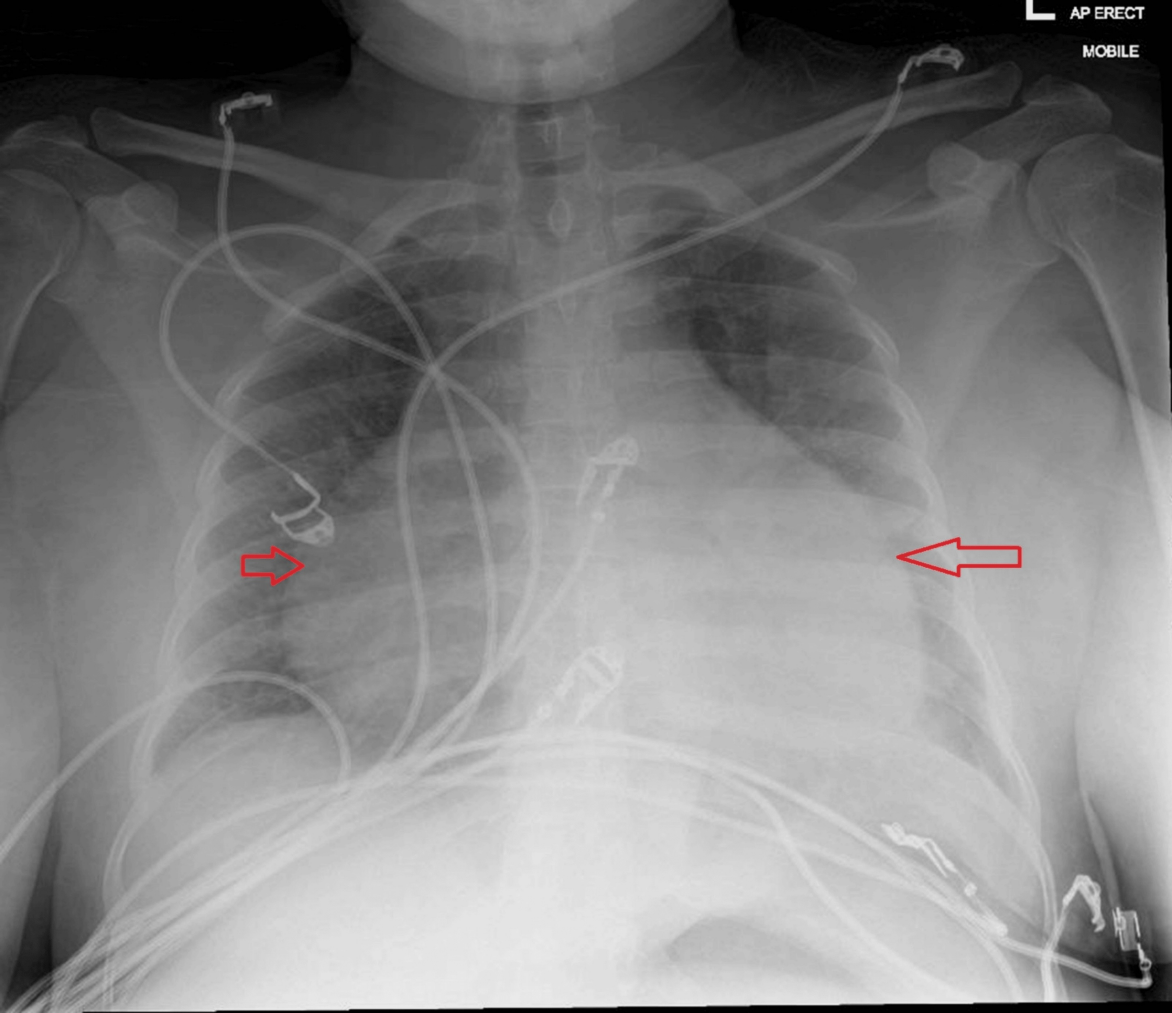
Repeat CXR on Day 22, during re-admission, demonstrating increased cardiothoracic ratio, indicates moderate to large pericardial effusion (red arrow) CXR, Chest X-ray

**Figure 9:**
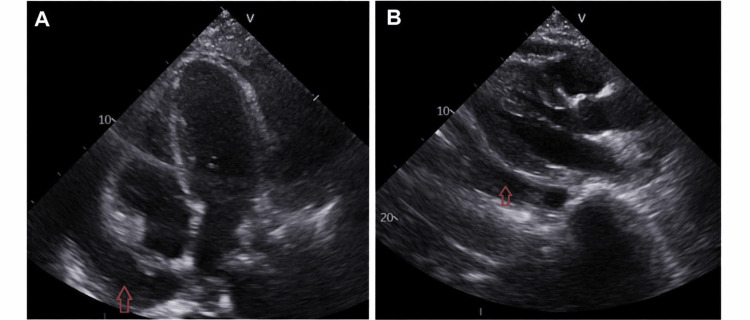
Transthoracic echocardiography findings (A) Parasternal long-axis view demonstrating pericardial effusion (red arrow). (B) Apical four-chamber view demonstrating circumferential pericardial effusion (red arrow).

**Figure 10:**
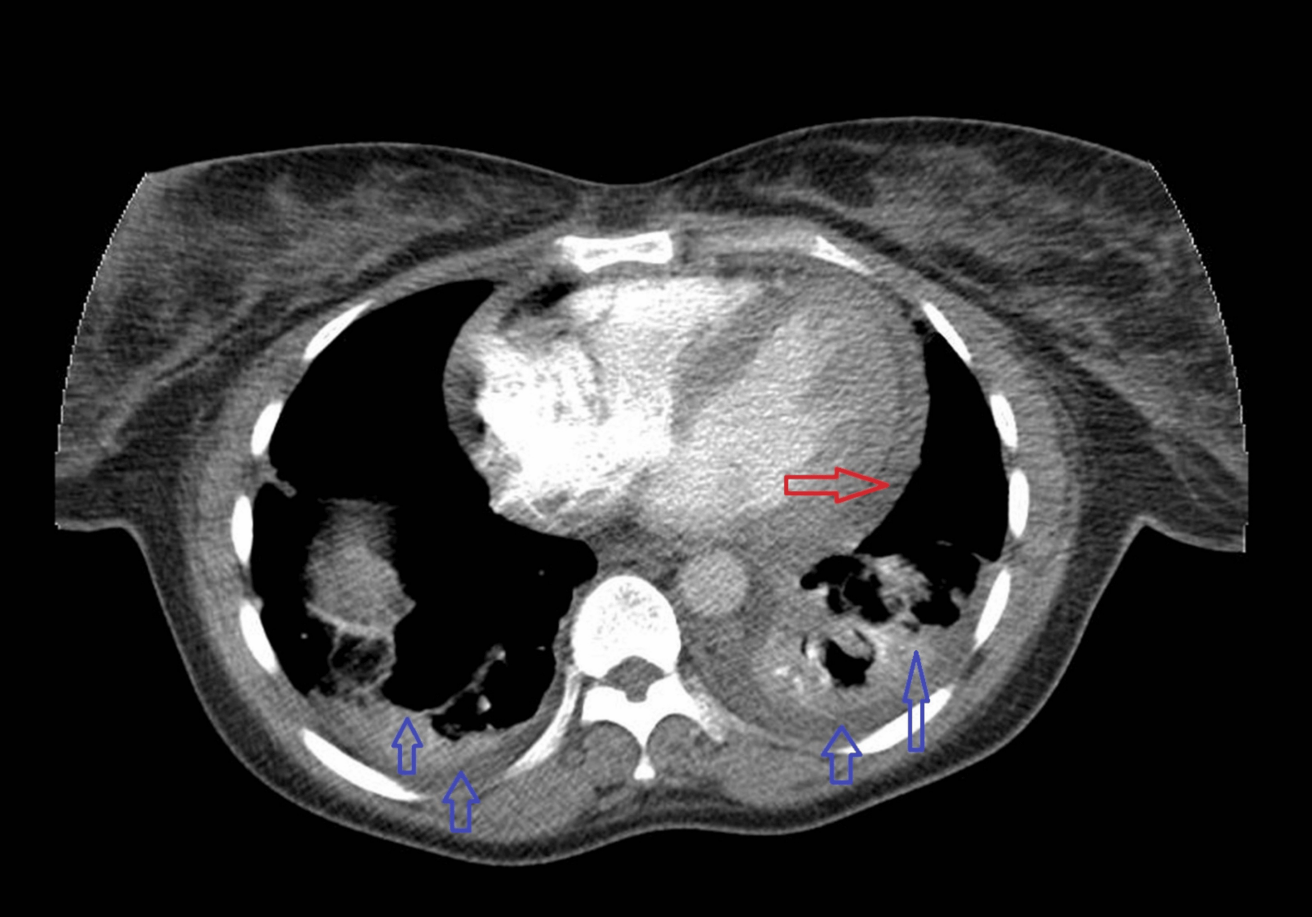
CT scan of the chest (CT pulmonary angiography) excludes pulmonary embolism, but shows mild B/L pleural effusion and B/L inflammatory changes in the lungs (blue arrows), along with mild pericardial effusion (red arrow) CT, Computed Tomography; B/L, Bilateral

During this admission, the patient also developed cardiac involvement, characterised by dynamic electrocardiographic (ECG) changes - likely widespread mild ST elevation (Figure 6) - along with high troponin (Figure 7), and moderate to large pericardial effusion on CXR (Figure 8). Echocardiogram (Figures 9A-9B) and computed tomography (CT) chest (Figure 10) confirmed pericardial effusion, which was ultimately suggestive of pericarditis/myopericarditis. Given clinical deterioration and an increasing HLH probability score, she was transferred to a tertiary HLH centre for specialist multidisciplinary management on Day 7 of admission.

At the tertiary centre, treatment included high-dose intravenous methylprednisolone, anakinra therapy, broad antimicrobial prophylaxis, and close multidisciplinary monitoring. She improved clinically, with falling ferritin levels (Figure 3) and haemodynamic stabilisation without organ support, and was discharged on oral corticosteroids, continuing anakinra on Day 13 of admission, with outpatient follow-up at the local rheumatology clinic one week later.

One week later, before attending the outpatient clinic, she was readmitted with chest pain, dyspnoea, and a mild pruritic rash on the face. Observations showed a high temperature, and further HLH blood tests showed evidence of increased CRP and ferritin levels compared to before. Imaging demonstrated an increase in pericardial effusion, as confirmed by CXR (Figure 8), and worsening cardiomegaly. An urgent subsequent echocardiogram showed moderate pericardial effusion (Figures 9A-9B). All these findings were consistent with an HLH flare, likely related to quick steroid tapering, prompting escalation of corticosteroid and anakinra dosing. Eventually, the patient demonstrated clinical stability with no new symptoms or deterioration. Biochemical markers remained stable and within acceptable ranges. The timeline of clinical events is shown in Table 3.

**Table 3:** Timeline of clinical events URTI, Upper Respiratory Tract Infection; CRP, C-Reactive Protein; HLH, Haemophagocytic Lymphohistiocytosis

Timepoint	Clinical Events									
Week -3 to admission	Sore throat, dysphagia, fever, rash, and polyarthralgia; treated with oral amoxicillin in the community									
Day 0 (admission)	Tachycardia, fever, and synovitis with elevated inflammatory markers and mild liver enzyme derangement; suspected URTI or reactive arthritis									
Days 1-3	Negative infectious and rheumatologic workup; persistent fever, rash, synovitis, and rising CRP and ferritin									
Day 4	Rheumatology review raised suspicion of adult-onset Still’s disease with secondary HLH									
Day 6	Initiation of intravenous methylprednisolone and anakinra after multidisciplinary discussion									
Day 7	Cardiac involvement (pericardial effusion with suspected myopericarditis); transfer to tertiary HLH centre									
Days 7-13	Clinical improvement with falling ferritin and stabilisation; discharged on oral steroids and anakinra									
One week post-discharge	Readmission with chest pain, dyspnoea, and fever; rising CRP and ferritin with worsening pericardial effusion									
After treatment escalation	Increased steroid and anakinra dosing with subsequent clinical stabilisation									

## Discussion

AOSD is a systemic inflammatory disorder of uncertain aetiology. It classically presents with high-spiking fever (>39 °C), an evanescent rash, arthritis or arthralgia, hyperferritinaemia, and potential multiorgan involvement [4,12,13]. In addition to the typical rash, a variety of atypical cutaneous manifestations - including urticarial eruptions, diffuse pruritus, plaques, and pustular lesions - have also been reported in association with AOSD [4,14]. Because of its rarity and heterogeneous presentation, establishing the diagnosis can be challenging.

AOSD demonstrates a bimodal age distribution, most commonly affecting individuals between 15-25 years and 36-46 years of age. The reported incidence ranges from approximately 0.16 to 0.4 cases per 100,000 persons, with an estimated prevalence of 1-34 cases per million, affecting males and females equally [4,12]. The pathogenesis of AOSD remains incompletely understood compared with other rheumatologic diseases. Emerging evidence suggests involvement of both autoinflammatory and autoimmune mechanisms, with dysregulated activation of pro-inflammatory cytokines - such as interleukin (IL)-1, IL-6, IL-8, IL-18, interferon-γ, and tumour necrosis factor-α (TNF-α) - playing central roles [1,12-15]. Although AOSD is considered an acquired condition, genetic susceptibility and familial clustering have been explored, and infectious triggers - both viral and bacterial - have been proposed. Ultimately, AOSD remains a diagnosis of exclusion, after ruling out infectious, malignant, and alternative autoimmune aetiologies [4,12,13]. Serologic markers, such as ANA and RF, are typically negative, though positivity may be observed in a small minority of patients [12,16]. Diagnosis is primarily clinical and most commonly guided by the Yamaguchi criteria (Table 3), which include four major features (fever, arthralgia, characteristic rash, and leukocytosis) and five minor features (sore throat, lymphadenopathy or splenomegaly, liver dysfunction, and negative ANA and RF) [4,9,10]. A diagnosis requires at least five criteria, including two major criteria. In the present case, the patient fulfilled the major criteria of fever and leukocytosis, along with minor criteria including splenomegaly, elevated liver enzymes, and negative ANA and RF, after exclusion of other causes of transaminitis. MAS represents one of the most severe haematologic complications associated with AOSD, occurring in approximately 10%-15% of affected individuals.

MAS is characterised by an uncontrolled hyperinflammatory response, leading to cytokine storm, coagulopathy, haemophagocytosis, and progressive organ dysfunction. Mortality rates in rheumatologic-associated MAS have been reported to range from 30% to 40%. MAS has also been described in association with systemic juvenile idiopathic arthritis, systemic lupus erythematosus, haematologic malignancies, solid tumours, and various infectious triggers, including viral, bacterial, fungal, and parasitic pathogens. Presentation of AOSD during pregnancy is rare, and MAS as the initial manifestation in pregnancy is even more uncommon [4,12,17-19]. HLH is a rare but life-threatening immunologic syndrome, marked by uncontrolled activation of cytotoxic lymphocytes and macrophages, resulting in cytokine-mediated tissue injury and multiorgan dysfunction [19]. HLH may be familial or secondary, with secondary HLH typically triggered by infection, malignancy, or chronic inflammatory disease. Early recognition and prompt treatment are therefore essential for survival. Common laboratory abnormalities include cytopenias; markedly elevated ferritin; increased liver enzymes; elevated lactate dehydrogenase and triglycerides; elevated D-dimer and soluble IL-2 receptor α (sCD25); and reduced fibrinogen levels [4,20].

Diagnostic frameworks such as the HLH-94 and HLH-2004 criteria were originally developed for paediatric familial HLH but are widely applied in adult and secondary forms due to the absence of better validated tools. According to the HLH-2004 criteria, diagnosis requires fulfilment of at least five of eight clinical and laboratory features [9-12]. Management of secondary HLH focuses on the identification and treatment of the underlying trigger, alongside immunosuppressive therapy. Current therapeutic strategies commonly include high-dose intravenous corticosteroids, IL-1-targeted biologic therapy, and cyclosporine A. Despite therapeutic advances over recent decades, mortality - particularly in adult HLH - remains substantial [4,12,17,19].

In this case, the constellation of feverish illness, sore throat, rash, arthritis, and hyperferritinaemia was consistent with AOSD, which subsequently progressed to secondary HLH, a recognised and life-threatening complication. Pregnancy-associated HLH is rare but associated with high maternal and foetal morbidity. Reported therapies include corticosteroids, intravenous immunoglobulin, cyclosporine, and biologic therapy such as anakinra, with increasing evidence supporting the safety and effectiveness of IL-1 blockade during pregnancy when maternal benefit outweighs foetal risk [4,8,14-16]. Pericardial effusion and myopericarditis are uncommon manifestations of HLH but reflect severe cytokine-mediated systemic inflammation, highlighting the importance of early cardiac evaluation in deteriorating patients [21].

Comparison with previously reported cases

AOSD complicated by secondary HLH during pregnancy is exceedingly rare, with only a limited number of cases described in the literature. Similar to previously reported patients [4,14,16], our case demonstrated persistent high-grade fever, markedly elevated inflammatory markers, hyperferritinaemia, liver dysfunction, and cytopenia, all of which raised concern for MAS/HLH. However, unlike many reported cases, where systemic symptoms or arthritis predominate at presentation, this patient initially presented with features suggestive of a URTI, contributing to diagnostic delay. Cardiac involvement, as observed in our patient, has been inconsistently reported and may indicate severe systemic inflammation. Consistent with prior literature, early recognition and prompt initiation of immunosuppressive therapy were critical for clinical improvement and prevention of maternal and foetal complications [14,16]. This case, therefore, reinforces the importance of considering AOSD-associated HLH in pregnant patients presenting with unexplained fever, extreme hyperferritinaemia, and multiorgan involvement despite negative infectious workup.

Clinical implications

This case highlights several important clinical considerations in the recognition and management of AOSD complicated by secondary HLH during pregnancy. First, persistent high-grade fever, with markedly elevated inflammatory markers and hyperferritinaemia in the absence of identifiable infection, should prompt early evaluation for autoinflammatory or rheumatologic disorders, including AOSD and MAS. Second, atypical initial presentations, such as symptoms resembling a URTI, may delay diagnosis and increase the risk of progression to life-threatening systemic inflammation and multiorgan involvement. Third, pregnancy adds diagnostic and therapeutic complexity because clinical features may overlap with obstetric conditions, and treatment options must balance maternal benefit with foetal safety. Early multidisciplinary involvement and timely initiation of targeted immunosuppressive therapy - particularly corticosteroids and IL-1-directed treatment - are crucial to reduce morbidity and mortality. Greater awareness of this rare but severe presentation may facilitate earlier diagnosis and improve maternal and foetal outcomes.

## Conclusions

AOSD occurring during pregnancy may be associated with adverse maternal and foetal outcomes, particularly when complicated by MAS. The coexistence of active AOSD and MAS further amplifies clinical risk and presents significant therapeutic challenges, largely due to the limited evidence regarding the safety and efficacy of available treatments in pregnancy. This case highlights the life-threatening and diagnostically complex course of AOSD complicated by secondary HLH in pregnancy, initially mimicking a URTI before progressing to severe systemic hyperinflammation with cardiac involvement.

Prompt recognition and early initiation of appropriate immunosuppressive therapy are crucial to optimise outcomes for both the mother and the foetus, and IL-1-targeted therapy represents a reasonable and potentially effective treatment strategy in pregnant patients with AOSD-associated MAS. Increased clinical awareness of HLH complicating AOSD in pregnant individuals presenting with unexplained hyperferritinaemia and systemic inflammation may facilitate earlier diagnosis and improved maternal outcomes. We anticipate that this case description will contribute to improved recognition and management of this rare but serious presentation of MAS in pregnancy associated with new-onset AOSD following a URTI.
